# Is the commercial determinants conversation confined to the health sciences? Potentially, and that’s a problem

**DOI:** 10.1186/s12992-023-00989-8

**Published:** 2024-01-02

**Authors:** Luc Louis Hagenaars, Nason Maani, Laura Anne Schmidt

**Affiliations:** 1 University of California San Francisco School of Medicine, Philip R. Lee Institute for Health Policy Studies, Campus Box 0936, 490 Illinois Street, Floor 7, San Francisco, CA 94158 USA; 2 Department of Public Health, Amsterdam UMC Location AMC, Meibergdreef 9, Amsterdam, 1105 AZ the Netherlands; 3 Global Health Policy Unit, School of Social and Political Science, University of Edinburgh, 15a George Square, Edinburgh, UK

**Keywords:** Meta research, Commerce, Medicine, Citation analysis, Trade

## Abstract

The commercial determinants of health (CDoH) are attracting increased interest and are of great importance when discussing how trade affects health. Through a citation analysis of recent foundational CDoH documents (a Lancet paper series and an Oxford University textbook), we find that fully 71% of all citations reference the health sciences. The health sciences may be well suited to documenting the specific pathways of how commercial (by)products and practices harm human health. However, to operationalize upstream solutions for mitigating these harms, our citation analysis suggests that the field can engage political scientists, economists, sociologists, the trade law and business, as well as advocates in civil society and journalism, more so than it currently does. With CDoH explicitly referring to the interaction between commerce and health, CDoH researchers might be uniquely positioned to get health on the agenda of others, which requires that CDoH methods, datasets, evidence reviews, and proposed interventions are drawn from the widest possible range of sources.

**Supplementary Information:**

The online version contains supplementary material available at 10.1186/s12992-023-00989-8.

## Background

The relatively young concept of commercial determinants of health (CDoH) is attracting increased interest with the recent publication of a Lancet series on the topic [1–3], and a dedicated textbook by Oxford University Press [4]. There is a World Health Organization factsheet on CDoH, outlining the direct and indirect effects of such actors across a range of environments and health conditions, and noting that effective public health actions can respond to such determinants [5]. The CDoH have been defined in a range of ways [5], with the Lancet series defining them as “the systems, practices, and pathways through which commercial actors drive health and equity” [1]. The growing interest in this field may reflect that the cumulative effects of commercial activity are arguably the greatest threat to human and planetary health of the 21st century. It is for this reason it is critical that groundwork for CDoH scholarship as a field is inclusive, rather than it becoming a new silo within health sciences [4].

### Citation analysis

**Fig. 1:**
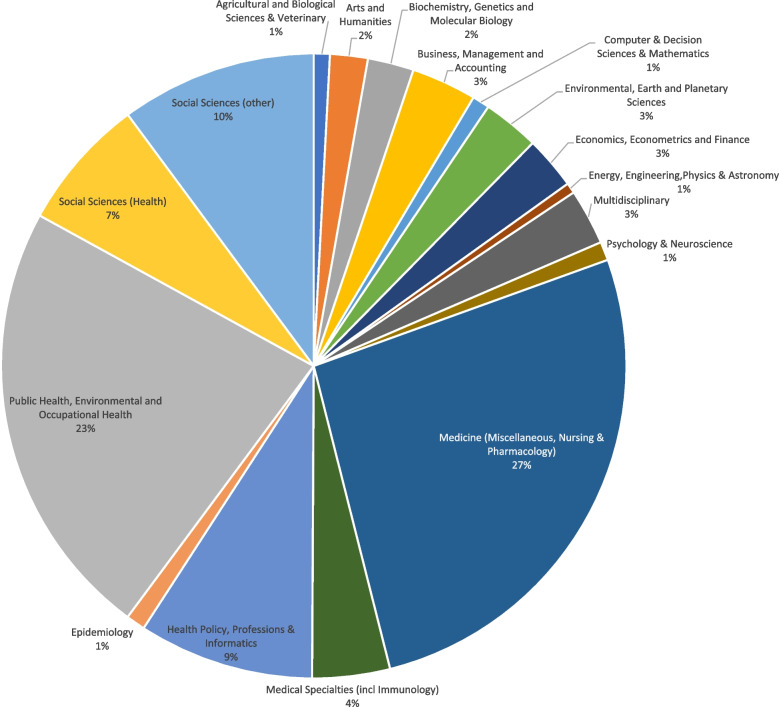
Disciplines cited in Oxford University Press texbook and Lancet series on Commercial Determinants of Health

To define the boundaries around scholarship under the new header of CDoH, we conducted a citation analysis of journals referenced in the recent Lancet Commission series and Oxford University Press volume using the SCImago database [6] (see Additional file 1 for methods and original data). Through this, we can provide a snapshot of where these CDoH scene-setting outputs ‘sit’ within the scientific universe. As Fig. 1 shows, CDoH researchers cite publications from a wide variety of disciplines, with medicine (27%) and public health (23%) being the most cited. However, overall, 64% of citations reference medicine-oriented sources, including the medical specialties, health policy, and epidemiology. If health-oriented social science journals are included, then fully 71% of all citations reference the broad spectrum of health sciences. Results are similar for the Lancet series and Oxford University Press textbook [1–4]. *Globalization and Health* is the most cited academic journal.

### Engaging more with non-health disciplines

What should we make of this? Given that CDOH research focuses on “the systems, practices, and pathways through which commercial actors drive health and equity” [1], practical solutions to these health harms will inevitably come from the governmental, legal, trade, business, and economic sectors of society. While it is heartening to see that there is a conscious effort to engage with these disciplines, there may be scope for much more, particularly among social sciences (10% of the time), and business and economics (3%) (see Fig. 1).

By focusing on individual and specific diseases and risk factors, the health sciences are well suited to documenting the specific pathways of how commercial (by)products and practices harm human health. But, for the same reasons, it needs engagement with other disciplines and civil society to operationalize upstream solutions to mitigate these harms. Our citation analysis, while limited to a small subset of outputs, suggests that scholars of the CDoH could do more around the edges of disciplines able to diagnosis and treat the problem: specialists trained to understand the wider effects and ecosystems in which companies and business sectors operate, the intergovernmental dynamics that drive trade, the incentive structure and culture of health-harming industries, and the social and political mobilizations required to effectively critique and bring about institutional change in political and economic systems [1, 2, 4].

The argument for greater collaboration between health and other disciplines has been made for decades under the header of ‘Health in All Policies’ [7]. This concept proves hard to implement, as other sectors tend to view health as the exclusive domain of medicine and public health, explaining why it might be difficult for CDoH researchers to publish in and cite from non-health journals. Greer et al. [7] propose a solution to these issues by emphasizing bidirectionality under the label ‘Health for All Policies’. Wealth and health together form a reinforcing feedback loop, but in the policy arena, health and the economy are often pitted against each other, with policymakers prioritizing short-term benefits for specific sectors over long-term economic benefits for society. The field of CDoH, with its explicit reference to the interplay between commerce and health, stands in a unique position to light the fact that health policies can significantly contribute to the overall economy, especially through regulating large but extractive health-harming industries with large externalities. Emphasizing that such regulations ultimately benefit the broader economy could serve as an avenue for CDoH research that is suitable for publication in health and non-health journals.

### Engaging other sources of evidence

Considering the multi-level influences of just a single large, multinational company across its corporate political activities, marketing, supply chain, products, employees and associated third parties, there is a clear need for triangulation across disciplines and evidence types in assessing its impacts on health. As such entities may both influence and be influenced by wider environments involving trade, law, governance, ethics, public discourse, and media, it is important that CDoH methods, datasets, evidence reviews, and proposed interventions are drawn from the widest possible range of sources [8]. CDoH researchers should also consider that academic evidence in and of itself rarely leads to action, but that this requires engagement with investigative journalism or civil society activism.

Another point of epistemic consideration is that most health-harming corporations are multinationals with head offices and shareholders based in high-income countries. These corporations are increasingly entering low-income and middle-income countries (LMICs), therefore, the burden of disease caused by their products and practices is also increasingly displaced upon LMICs. The young CDoH field should therefore avoid the types of epistemic injustices that scholars argue are in place in academic global health [9].

## Conclusions

As the CDoH field continues to grow and mature, the field should not just expand beyond the tobacco industry or closely related harmful products—something that all three of the recent agenda-setting publications discussed here have argued. From a consequentialist perspective, the next stage in the field’s development, would be a larger emphasis on seeking industry-specific and cross-industry evidence and interventions at the systems and societal levels. The health sciences alone will not identify these. It is important to be mindful of subdivisions in a field forming that prevent collective progress, as observed in One Health [10] or Global Health [9]. For the CDoH field to reach its full potential, CDoH researchers should engage more with political scientists, economists, sociologists, the trade law and business, as well as investigative journalists and advocates in civil society far beyond those traditionally linked to health.

### Supplementary Information

**Additional file 1.** Journal citations in Oxford University textbook and Lancet series on CDoH, grouped by academic discipline for crafting Fig. 1.

## Data Availability

All data generated or analyzed during this study are included in this published article [and its supplementary information files].

## Abbreviation

CDoH: Commercial Determinants of Health
